# Magnetic Molecularly Imprinted Polymers: An Update on Their Use in the Separation of Active Compounds from Natural Products

**DOI:** 10.3390/polym14071389

**Published:** 2022-03-29

**Authors:** Marisa Dwi Ariani, Ade Zuhrotun, Panagiotis Manesiotis, Aliya Nur Hasanah

**Affiliations:** 1 Pharmaceutical Analysis and Medicinal Chemistry Department, Faculty of Pharmacy, Universitas Padjadjaran, Sumedang, Bandung 45463, West Java, Indonesia; marisa13001@mail.unpad.ac.id; 2 Pharmacy Biology Department, Faculty of Pharmacy, Universitas Padjadjaran, Sumedang, Bandung 45463, West Java, Indonesia; ade.zuhrotun@unpad.ac.id; 3 School of Chemistry and Chemical Engineering, Queens University of Belfast, Belfast BT9 5AG, UK; p.manesiotis@qub.ac.uk; 4 Drug Development Study Center, Faculty of Pharmacy, Universitas Padjadjaran, Sumedang, Bandung 45463, West Java, Indonesia

**Keywords:** active compound separation, magnetic molecularly imprinted polymer, natural product

## Abstract

During the last few years, separation techniques using molecularly imprinted polymers (MIPs) have been developed, making breakthroughs using magnetic properties. Compared to conventional MIPs, magnetic molecularly imprinted polymers (MMIPs) have advantages in sample pretreatment due to their high specificity and selectivity towards analytes as a result of their larger specific surface areas and highly accessible specific binding sites. The techniques of isolation of active compounds from natural products usually require very long process times and low compound yields. When MMIPs are used in sample separation as Solid Phase Extraction (SPE) sorbents, the MMIPs are introduced into the dissolved sample and spread evenly, and they form bonds between the analyte and the MMIPs, which are then separated from the sample matrix using an external magnetic field. This process of separating analytes from the sample matrix makes the separation technique with MMIPs very simple and easy. This review discusses how to synthesize MMIPs, which factors must be considered in their synthesis, and their application in the separation of active compounds from natural products. MMIPs with magnetic core-shells made by co-precipitation can be a good choice for further development due to the high synthesis yield. Further optimization of the factors affecting the size and distribution of magnetic core-shell particles can obtain higher synthesis yields of MMIPs with higher adsorption capacity and selectivity. Thus, they can isolate target compounds from natural plants in high yields and purity.

## 1. Introduction

These days, plants are widely used by people in various parts of the world to meet their daily needs, such as medicines, food, beverages, and cosmetics. People consume natural products because of their known health-related benefits. In 2020, Allied Market Research predicted that the global natural product market for food and beverages will grow by about 13.7% per year in the future [1,2,3,4]. Plants are often used in medicines because people believe that diseases can be cured by using plants. Treatment using plants also has advantages in terms of toxicity and side effects compared to the use of modern medicines [5]. This is a factor in many studies conducted in the development of active plant compounds that can be used as medicines [6]. In fact, chemical compounds contained in natural products can be very complex and many derived compounds have been used in the discovery of new drug compounds, and 45% of best-selling drugs are derived from chemical compounds from natural products and their derivatives [7]. Some active plant compounds that have been used in medicines include paclitaxel and its derivatives, vincristine, vinblastine, camptothecin and its analogs, which are used as anticancer agents [8], ginkgo biloba, which is used as therapy in Alzheimer’s disease and vascular or mixed dementia [9], and fingolimod, which is used in anti-sclerosis [10]. However, the biggest challenge in research using plant materials is the difficulty of separating and isolating the active compounds. This is because each plant contains a large number of active compounds and the isolation of active compounds involves long stages, starting from plant collection, extraction, multiple-cycle fractionation, evaluation of extract and fraction bioactivity, and separation of single compounds. Each stage presents various obstacles, including very few isolates being obtained [11]. Kumar (2013) isolated, purified, and characterized vinblastine and vincristine from 1 L of Catharanthus roseus culture filtrate, obtaining vinblastine and vincristine in yields of 0.0000076% (76 μg) and 0.0000067% (67 μg) [12]. Hakim (2018) isolated ethyl p-methoxycinnamate from *Kaempferia galanga* L. and obtained 0.49 g crystalline ethyl p-methoxycinnamate from 50 g samples (0.98%) [13]. Lin (2015) also conducted research to isolate luteolin from 5.8 kg *Dendranthema morifolium* flowers and obtained 0.19% luteolin in the ethanolic extract [14]. Jokic (2016) isolated scopoletin from *Helichrysum italicum* using a supercritical CO_2_ extraction method and obtained the highest yields of 0.00001933% [15]. As a comparison, Wan et al. (2018) conducted a study on new dual-functional monomers based on imprinted polymers (MIPs) for the isolation of myricetin from Carthamus tinctorius L. and Abelmoschus manihot plants and obtained myricetin yields of 79.82% to 83.91% and 81.50% to 84.32%, respectively. Lai et al. (2002) also used MIPs as a Solid Phase Extraction (SPE) sorbent to isolate matrine from Sophora flavescens and obtained 71.4% matrine yields. Therefore, a specific and efficient method of separation and isolation of active plant compounds is required [16].

Molecularly imprinted polymers (MIPs) represent a technique for the design of novel materials that have specific template cavities that can recognise biological and other molecules, such as biological receptors, antibodies [17], foods [18], medicines [19], amino acids [20], and proteins [21]. Recently, MIPs have been used in various applications, such as artificial antibodies [22], catalysts [23], and biosensors [24] and in drug delivery [25], purification and separation science [26,27], chromatographic separation [28], and solid-phase extraction [18,29]. MIPs are used in affinity chromatography [30] as the stationary phase in high-performance liquid chromatography [30]. Among the many applications of MIPs for natural products, there are several polymerization techniques, namely bulk, in situ precipitation and suspension polymerization. The MIP polymerization technique used by researchers for natural product analysis is almost the same as the previously published MIP polymerization technique. All techniques have been widely applied to the separation or isolation of natural products, as can be seen in Table 1.

Table 1 shows that the most widely used polymerization methods are the bulk polymerization and precipitation methods. MIP particles obtained by bulk polymerization should be treated by many pretreatment steps, such as grinding and sieving procedures, before being used for separation and purification. The grinding and sieving steps cause the resulting polymer particles to become irregular in size, which results in decreased permeability of the MIPs particles [32,39]. Regular polymer particles can be obtained by precipitation polymerization, which allows the formation of imprinted polymer particles by the same reaction as that used in bulk polymerization but still has the weakness of requiring large quantities of porogen [42,43,44,45,46,47,48,49,50]. MIP particles obtained by suspension polymerization yield uniformly shaped polymer particles, but these still do not meet expectations in sample separation from complex matrixes [51,52].

Recently, researchers have been interested in magnetic molecularly imprinted polymers (MMIPs), which have several advantages over conventional MIPs derived from the use of an external magnet [53,54]. MMIPs have been applied to analyze and separate food, environmental and biological biomacromolecules, and antibiotics. MMIPs are a combination of MIPs and magnetic nanoparticles (MNPs) that provide a magnetic feature wherein MMIPs are added to a sample solution containing the target analyte and stirred to enhance the absorption of the analyte into the magnetic polymer. This combination improves the adsorption selectivity and avoids being time-consuming due to the centrifugation and filtration stages in the separation process. Various methods and techniques for the development of magnetic nanoparticles as MMIP cores have been carried out to obtain the desired particle size, distribution, and chemical/physical properties [53,54,55,56]. In addition, MMIPs tend to have lower costs because they can be reused after a few simple processes. Therefore, many researchers have started to explore the use of MMIPs [57].

Cheng et al. (2020) carried out the synthesis and characterization of MIPs for the extraction and determination of kaempferol from apple samples. The synthesis was carried out using a magnetic core-shell Fe_3_O_4_@SiO_2_, with kaempferol as the template, acrylamide as the monomer, and ethylene glycol dimethacrylate (EGDMA) as the crosslinker. These MMIPs showed an adsorptivity of 3.84 mg/g for kaempferol and adsorption equilibrium was reached in 50 min [58]. Sadegh et al. (2021) extracted harmaline from *Peganum harmala* using MMIPs with core-shell Fe_3_O_4_@SiO_2_, harmaline as the template, methacrylic acid as the monomer, and EGDMA as the crosslinker. The maximum adsorption capacity of the MMIPs was 45.31 mg/g and of Magnetic Molecularly Nob-Imprinted Polymers (MNIPs) this was 16.46 mg/g. These results indicate that MMIPs can be effective sorbents with high efficiency and capacity [59].

Until now, there have been review articles related to MMIPs for the separation of antibiotics [56], food and the environment [53], biomarkers [57], and biomacromolecules [54]; however, there are no review articles discussing the use of MMIPs to separate active substances from natural products, their synthesis, and which factors must be considered in their manufacture. Hence, this review will discuss how to synthesize MMIPs, which factors must be considered in their manufacture, and their application in the separation of active compounds from natural products.

## 2. Materials and Methods

**Figure 1:**
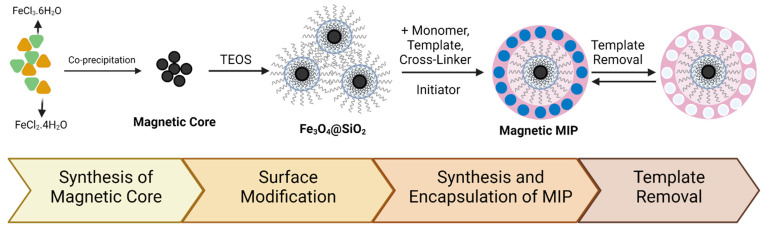
Schematic of MMIP synthesis.

An MMIP is a combination of magnetic particles with an MIP. Magnetic particles widely used in the synthesis of MMIPs are Ni [60], NiO [61], γ-Fe_2_O_3_ [61], and Fe_3_O_4_ [62,63,64,65]. The most commonly used magnetic particle is Fe_3_O_4_ due to its low toxicity and easy fabrication [66]. MMIP synthesis consists of several main steps: the first is the synthesis of the magnetic nanoparticles; the second is the modification of the magnetic core-shell surface; the final step is the synthesis of the MMIP itself [67]. A schematic of MMIP synthesis is presented in Figure 1.

### 2.1. Synthesis of Magnetic Nanoparticles for MMIPs

**Figure 2:**
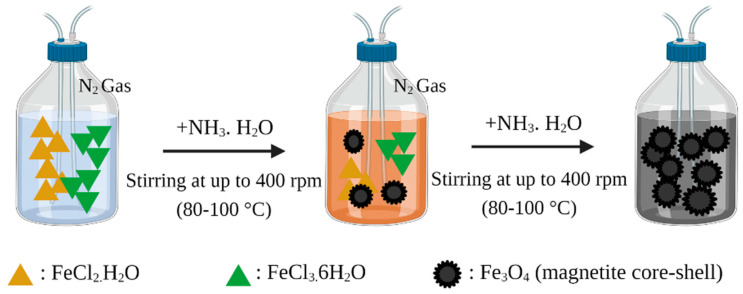
Synthesis of magnetic cores: co-precipitation method.

Magnetite (Fe_3_O_4_) nanoparticles are generally used as magnetic materials in the world of medicine and pharmaceuticals due to their biocompatibility, biodegradability, low toxicity, magnetic properties, and easy preparation. This magnetite preparation can be conducted by various techniques, namely co-precipitation, solvothermal/hydrothermal, oxidation method, flow injection synthesis, supercritical fluid method, microemulsion, thermal decomposition, chemical vapor deposition, electron beam lithography, microwave assisted, and sonochemical [58]. However, among all these Fe_3_O_4_ nanoparticle preparation techniques, the most commonly used techniques for magnetic preparation of MMIP nanoparticles are co-precipitation and solvothermal/hydrothermal techniques [54,56]. The first step in the manufacture of MMIPs is to make magnetite, with the final product being in the form of iron (II, III) oxide or ferrosoferric oxide (Fe_3_O_4_) [59]. The magnetite is obtained using co-precipitation (as shown in Figure 2) of hydrated iron (II) chloride (FeCl_2_·H_2_O) and iron (III) chloride (FeCl_3_·6H_2_O). Iron in the higher oxidation state can also be gained from iron (II) sulphate (FeSO_4_·7H_2_O). Both reactions are performed in sodium hydroxide or ammonia solution at the temperature range of 80–100 °C [60,61,62]. This co-precipitation is carried out with nitrogen gas flowing continuously while stirring is carried out at up to 400 rpm. A black precipitate will be obtained as the end product of the reaction, which is then collected using an external magnetic field. The final product is then washed with ultrapure water until the pH of the rinse water is neutral. In the final step, the black precipitate is dried for 24 h at 60 °C [63].

**Figure 3:**
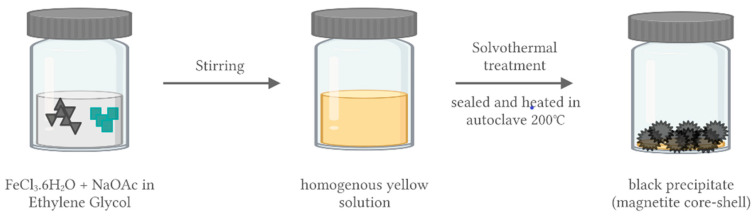
Synthesis of magnetic cores: solvothermal method.

The synthesis of the magnetic cores can be performed using the solvothermal method, as shown in Figure 3, following Shao et al. (2012). The procedure involves dissolving FeCl_3_·6H_2_O and sodium acetate in ethylene glycol with vigorous stirring. A homogeneous yellow solution is produced, which is then transferred to an autoclave, sealed, heated at 200 °C for 8 h, and then cooled to room temperature. The product of the reaction is black magnetite particles, which are then washed several times with ethanol and dried at 60 °C for 12 h [64].

Co-precipitation can produce magnetite in a high yield by an easily performed procedure. However, the shape and size of the particles produced by this method are irregular. The solvothermal method has resulted in a more uniform size and distribution of Fe_3_O_4_ magnetite particles. However, the method involves higher cost and greater effort due to the stage of heating to very high temperatures [64].

### 2.2. Modification of Magnetic Core-Shell Surface

**Figure 4:**
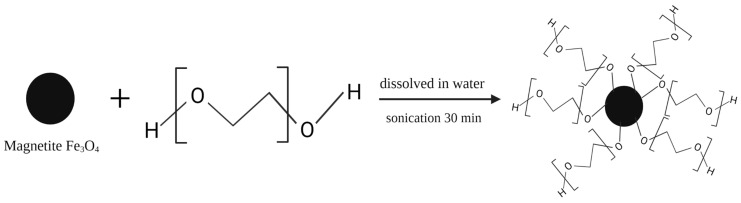
Surface modification using polyethylene glycol (PEG).

After obtaining Fe_3_O_4_ magnetite, it was then coated with a SiO_2_ film by hydrolysis with tetraethyl orthosilicate (TEOS). The procedure used is as follows: Fe_3_O_4_ is evenly dispersed in 80% ethanol and ultrasonicated for 30 min, with ammonium hydroxide and TEOS added dropwise to the solution. Then, the mixture is stirred for 12 h at 500 rpm at 45 °C. The Fe_3_O_4_@SiO_2_ produced after stirring is collected with the help of an external magnet and then washed several times with 95% ethanol and dried for 12 h at 60 °C [65]. This modification of the magnetite surface aims to protect magnetite from the oxidation or dissolution that can occur in the following stage of the MMIP formation reaction [66] and modification with SiO_2_ can also increase the dispersion of magnetite in water and prevent agglomeration [67]. In addition, the presence of the SiO_2_ shell allows further modification of the magnetite. An example is the addition of a modification with 3-(trimethoxysilyl) propyl methacrylate (MPS) (Fe_3_O_4_@SiO_2_@MPS), which imparts a vinyl group to the magnetite surface, providing a binding site for further polymerization [68]. Graphene oxide (Fe_3_O_4_@SiO_2_-GO@MPS) [69] and triethylamine (Fe_3_O_4_@SiO_2_-CH=CH_2_) [63] can be an option to provide binding sites on the SiO_2_ shell. SiO_2_ is the most frequently used layer [66], but several other layers can also be used. Polychloromethylstyrene (PCMS) can protect the magnetic core-shell from corrosion under acidic and basic conditions [70], oleic acid (OA) can increase hydrophobicity [71], and Chen et al. (2012) also increased hydrophobicity by using polyethylene glycol (PEG) [72]. Figure 4 shows the surface modification by PEG.

### 2.3. Synthesis of MMIPs

**Figure 5:**
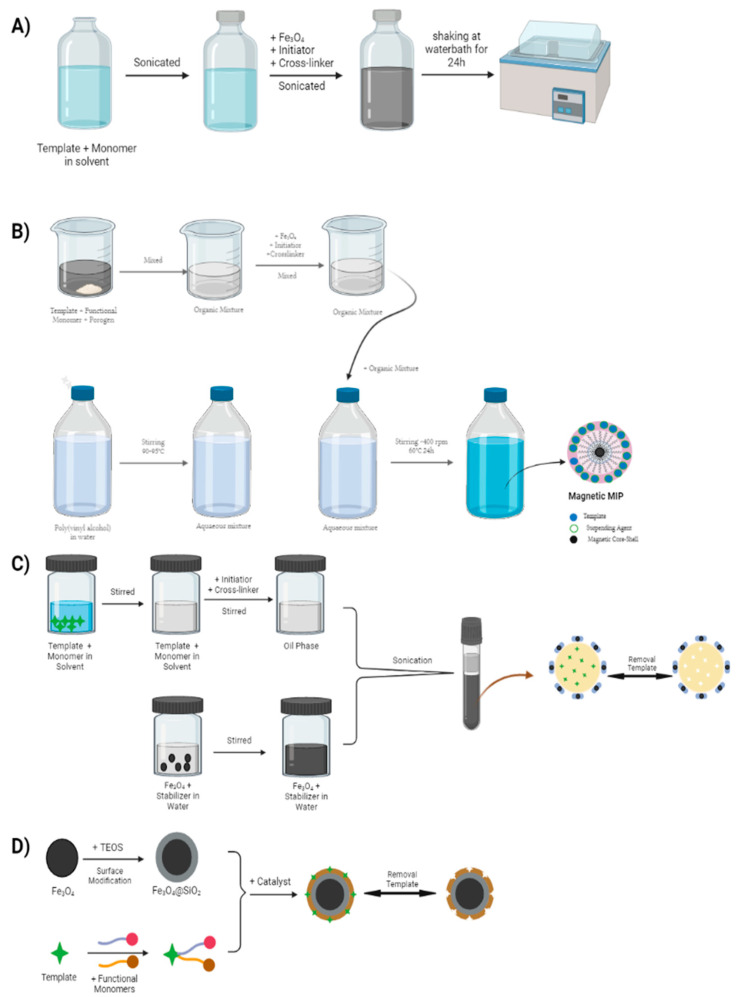
(**A**) Precipitation polymerization; (**B**) suspension polymerization; (**C**) emulsion polymerization; (**D**) sol–gel polymerization.

The final step is the synthesis of MMIPs, which can be carried out by two polymerization methods: free-radical polymerization and sol–gel polymerization. In free-radical polymerization, there is a reaction between template, functional monomer, and crosslinker in porogen solution, and the mixture is then added to the magnetic core-shell [73]. The polymerization process will start with the addition of an initiator in the form of free radicals, assisted by heating and irradiation [74]. Free-radical polymerization can be carried out using several different techniques, such as precipitation polymerization [75], suspension polymerization [76], and emulsion polymerization [77]. The method has a weakness in the use of highly toxic solvents such as toluene, chloroform, and acetonitrile, which can have adverse effects if they accumulate in the body and can also damage the environment. Therefore, an alternative method was developed using solvents that are not harmful to the body or the environment, such as water, i.e., the sol–gel polymerization method. This method also has the advantage of a very easy fabrication procedure compared to that of free-radical polymerization [73]. In the sol–gel polymerization method, the step begins with the hydrolysis of a precursor such as tetramethoxysilane [78], tetraethoxysilane [79], or other tetra-alkoxysilanes [80], which are then gradually condensed to form a gel (crosslinked silica materials) [81]. The polymer produced from the sol–gel method is very rigid because of the short distance between the crosslinking sites, meaning that the crosslinks formed in the polymer are very strong. The strong crosslinking is another advantage of sol–gel polymerization precipitates compared to free-radical polymerization because sol–gel polymerization precipitates have high stability and are resistant to swelling due to the strong bonds between the crosslinked sites. Therefore, sol–gel polymerization can produce MMIPs that can better maintain the shape and size of the specific sites for binding to the analyte [81,82]. Figure 5 shows the various polymerization methods.

### 2.4. Factors Affecting the Synthesis of MMIPs

MMIP synthesis was carried out in three main stages, namely the manufacture of magnetic cores, magnetic coating of the core-shell, and synthesis with MMIPs. Each stage has factors that can affect the process and the resulting MMIPs.

#### 2.4.1. Factors Affecting the Synthesis of Magnetic Nanoparticle Fe_3_O_4_

The size, shape, and particle distribution of the magnetic core strongly affect the success and effectiveness of MMIPs. Several factors can affect the particle size in the synthesis of the magnetic core. Mahajan et al. [83] conducted a study to compare the performance of magnetic nanoparticles produced by various polymerization methods. Those produced using co-precipitation polymerization were in very good yields, but the resulting particle shape and size distribution were irregular. This is in contrast to the magnetic nanoparticles produced by solvothermal polymerization, which showed better particle shape and size distribution. However, this method had the disadvantage of requiring a very long time and the use of very high temperatures [84]. As shown in Table 2, several factors in the synthesis of the magnetic core affect particle shape and size distribution.

In Yan’s research [84], different synthesis times and molar ratios of FeCl_3_ and protective agents were used in MMIP synthesis to investigate the effect on particle size. The synthesis was carried out at a temperature of 180 °C with reaction times of 24 h and 6 h, which produced particles of 50 nm and 20 nm, respectively. This shows that the shorter the reaction time, the smaller the particle size. In addition to reaction time, the molar ratio of FeCl_3_ and protective agents also affected the particle size. It was found that the particle size reduced (50 nm, 30 nm, and 20 nm) with the increase in molar ratio (4:3, 5:3, and 6:3, respectively). Saragi et al. studied the effect of reaction temperature on the synthesis of Fe_3_O_4_ by the co-precipitation method. The synthesis was carried out for 1 h with temperature variations of 25 °C, 40 °C, 60 °C, and 80 °C, which produced particles of 10.14 nm, 10.32 nm, 10.95 nm, and 11.66 nm. The higher the reaction temperature, the smaller the particle size.

Wu et al. [86] concluded that the shape and size of the magnetic nanoparticles produced depend on the type of salt used, the ratio of ferric to ferrous salt, the reaction temperature, the pH of the solution, and the ionic strength of the medium. Different reaction temperatures can cause the reaction to be imperfect, resulting in the size and shape of the resulting nanoparticles becoming irregular. Fe_3_O_4_ nanoparticles are very unstable at temperatures below ambient, and if the synthesis process takes place below ambient temperature, the Fe_3_O_4_ is easily oxidized to Fe_2_O_3_ [87]. In addition, magnetic Fe_3_O_4_ nanoparticles can easily be dissolved in acidic conditions. To maintain the stability of the magnetic nanoparticles that are produced, the reaction must be carried out under anaerobic conditions to prevent the oxidation of Fe_3_O_4_ by oxygen in the air.

#### 2.4.2. Factors Affecting the Synthesis of Magnetic Core-Shell

Magnetic nanoparticles have high magnetic properties; however, these magnetic nanoparticles have some drawbacks, such as lack of biocompatibility with targets and poor solubility in water. To overcome these shortcomings, magnetic nanoparticles are modified by coating magnetic nanoparticles using coating materials such as organic materials (polymers and surfactants) and inorganic materials (silica, metals, carbon). Zhong et al. (2008) carried out magnetic modification of nanoparticles coated with silica (SiO_2_) through the hydrolysis of TEOS. The Fe_3_O_4_ nanoparticles obtained were dispersed in 200 mL of 80% ethanol and ultrasonicated for 30 min. Then, 5 mL of ammonium hydroxide and 0.6 mL of TEOS were added to the mixture dropwise. The mixture was mechanically continuously stirred at 500 rpm for 12 h at 45 °C. The Fe_3_O_4_@SiO_2_ nanoparticles obtained by magnetic separation were then washed with water three times for neutralization and dried in an oven at 60 °C for 12 h [65].

Based on the procedure carried out in the magnetic modification stage of these nanoparticles for becoming a magnetic core-shell, several factors can affect the results of the magnetic core-shell synthesis, such as stirring, sonication, washing, drying, reaction time, and temperature. There has been no further research related to the variations on the factors of this core-shell magnetic synthesis; hence, further optimization is needed to develop a more effective and efficient core-shell magnetic modification.

#### 2.4.3. Factor Affecting the Synthesis of MIPs

The MMIP synthesis was carried out by reacting the modified magnetic core-shell with MIP components, namely a template, functional monomer, crosslinker, and solvent. Before reacting the magnetic core-shell, the step begins with pre-polymerization between the template and the functional monomer. The stronger the interaction between the template and the functional monomer, the more stably the template–monomer complex will be formed with a high imprinting factor. The amount of functional monomer used can also affect the binding capacity between monomer and template. The study of Zhao et al. [88] showed the variation of methyl methacrylate (MMA) monomer to MIP Solasenol, where the highest adsorption capacity of 43 mg/g was obtained at a 0.2 mmol MMA concentration. When varying the concentration of MMA used, 0.25 mmol, 0.30 mmol, and 0.35 mmol, the adsorption capacity decreased 41 mg/g, 37 mg/g, and 36 mg/g, respectively. The type of template–monomer bond is also a factor in the selection of functional monomers to form the active site in the MIP. The research of Baros et al. [89] showed that the hydrochlorothiazide template had three interaction sides, thus the functional monomer was selected based on the interaction energy formed between the monomer and hydrochlorothiazide through computational simulations, with the highest bond energy being methacrylic acid monomer through hydrogen bonds.

The next component that affects the synthesis of MIPs is the crosslinker. Crosslinkers have an important role in MIP formation by influencing morphological characteristics, durability, optimal stiffness, and three-dimensional structure. The higher number of crosslinkers makes it possible to obtain stable porous materials. However, the increasing number of crosslinkers can also increase the pore diameter size of the MIPs, thereby reducing the binding capacity of MIPs. In addition, the number of crosslinkers also affects the adsorption performance of MIPs. The study of Zhao et al. [88] showed that the adsorption performance increased with the increase in the number of crosslinkers. Moreover, after reaching the optimum condition, the increase in crosslinkers no longer affects the adsorption performance, and there is even a possibility of decreasing the adsorption performance of MIPs.

The solvent has a very important role in the synthesis of MIPs. The solvent must be able to dissolve all synthesis materials without disturbing the polymerization process. MIP synthesis is usually carried out in organic solvents to enhance electrostatic interactions and hydrogen bonding between templates and functional monomers. Polar solvents will affect the template–monomer interaction more than semipolar solvents. The research of Song et al. [90] compared various porogens for the synthesis of MIP Quercetin with acrylamide monomers such as 1,4-dioxane, tetrahydrofuran (THF), acetone, and acetonitrile (from lowest to highest polarity). In acetonitrile, the solvent interacts too strongly with the template and monomer, causing the formation of the template–monomer complex to be difficult to form. In 1,4-dioxane (less polar), the formation of the template–monomer complex is very strongly bound, but the polymer solubility is very low in less polar solvents, meaning that MIP will precipitate quickly. In THF (semipolar) solvents, the formation of the template–monomer complex becomes optimum because the interaction of THF with the template or monomer is no more subtle than the interaction between the template and the monomer.

## 3. Characterization of MMIPs

In general, the physical characterization of MMIPs is the same as that for MIPs. However, there are some additional evaluations in MMIPs. The characterizations carried out are shown in Table 3.

SEM, FT-IR, and BET were used to observe the physical properties, morphology, and porosity of MMIPs. Foroughirad et al. (2018) synthesized MMIPs with Fe_3_O_4_@SiO_2_-APTES magnetic material and analyzed the functional groups found in MMIP, MNIP, Fe_3_O_4_@SiO_2_, and Fe_3_O_4_@SiO_2_-APTES using FT-IR. There were characteristic peaks of the stretching vibration of Si-O-Si and Si-OH at 1103 cm^−1^ and 954 cm^−1^, respectively, and of Si-O’s vibration at 794 cm^−1^ and 470 cm^−1^. Specific surface area and porosity analysis was carried out using the BET method for MMIP and MNIP. A larger surface area will result in more porosity and active sites. The results of the SEM analysis show the morphology and size of the polymer particles. The diameter of the Fe_3_O_4_ magnetic core is about 335 nm, and the presence of surface modification increases the diameter by 40 nm [91].

XRD is used to analyze the diffraction spectroscopy of materials. Generally, a wide-angle XRD pattern indicates the presence of magnetic solids and a low-angle pattern indicates the pore structure of porous magnetic materials [94]. The XRD results observe the shape of the Fe_3_O_4_ diffraction peak and compare this with the standard Fe_3_O_4_ diffraction form. Normally, the magnetite structure has six characteristic diffraction peaks at 2θ = 30.12°, 35.35°, 43.08°, 53.48°, 57.92°, and 63.98°, which correspond to the (220), (311), (400), (422), (511), and (440) planes of Fe_3_O_4_, respectively. The presence of a diffraction peak at that point indicates that the Fe_3_O_4_ magnetite is still present in the MMIPs and has not been damaged during the synthesis and purification process. The absence of additional peaks other than those of Fe_3_O_4_ indicates that there is no contamination in the system [83,95].

In research by Xie et al. [63], protocatechuic acid (PA) was extracted from plant extracts using MMIP-SPE (Magnetic Molecularly Imprinted Polymer–Solid Phase Extraction). The synthesis of MMIPs was carried out by precipitation polymerization with a composition consisting of Fe_3_O_4_@SiO_2_ as the magnetic core, PA as the template, acrylamide as the monomer, EGDMA as the crosslinker, and AIBN as the initiator. The results of the synthesis were then characterized by XRD to study the stability or integrity of the Fe_3_O_4_ magnetic core, the results of which showed six characteristic peaks in the 2θ range of 20–70° (220, 311, 400, 422, 511, 440). The results obtained followed those of magnetite in the Joint Committee on Powder Diffraction Standards (JCPDS) database (JCPDS card: 1 9 6 29), indicating that Fe_3_O_4_ had not changed after the synthesis and purification process. Cheng et al. [96] also carried out the synthesis and characterization of MMIPs for the selective recognition and determination of quercetin from apple juice samples. XRD was used in the characterization and showed six characteristic 2θ peaks in the range 10–80°, corresponding to (220), (311), (400), (422), (511), and (440).

TGA evaluated the stability of the magnetic core encapsulated with the imprinted polymer by analyzing the change in polymer weight on heating. Xie et al. [63] synthesized MMIPs with Fe_3_O_4_@SiO_2_ magnetic material and evaluated them by TGA to measure the encapsulation. The analysis results showed that Fe_3_O_4_@SiO_2_ at a temperature of <200 °C experienced a weight loss of 5% due to water elimination. Meanwhile, MMIPs and MNIPs experienced a significant decrease in weight, of around 11% and 14%, respectively. The difference in weight loss of MMIPs and MNIPs on heating can be assumed to be due to differences in grafting template density in the MMIPs. A study by Kang [68] used the same magnetic material and analyzed Fe_3_O_4_@SiO_2_ and MMIPs. The weight of Fe_3_O_4_@SiO_2_ and the MMIPs decreased by 10.8% at a temperature of 20–400 °C. MMIPs experience extreme weight loss at 400–450 °C due to the decomposition of the molecularly imprinted layer.

The magnetic hysteresis loop shows the relationship between magnetic flux density and magnetic field strength. Magnetic hysteresis loop values can be used to observe the magnetic separation ability of MMIPs, evaluated using vibration sample magnetometry (VSM). In the study by Sadegh [97], MMIPs were synthesized for harmaline extraction using Fe_3_O_4_@SiO_2_-NH_2_. These MMIPs needed to have sufficient magnetic property so that a good separation of sorbent and analyte could be carried out. Sadegh used VSM to study the magnetic properties, and the results for Fe_3_O_4_, Fe_3_O_4_@SiO_2_, Fe_3_O_4_@SiO_2_-MNIP, and Fe_3_O_4_@SiO_2_-MMIP were 48, 33, 20, and 20 emu/g, respectively. From the results obtained, it can be seen that there was a decrease in the value of saturation magnetization after modification from MNIPs to MMIPs. The saturation magnetization value decreases due to the addition of properties to the magnetic core-shell, such as modifications with SiO_2_ and imprinted polymer.

Cheng [98] also evaluated the magnetic separation capability of MMIPs synthesized with Fe_3_O_4_@SiO_2_ and MIPs. The saturation magnetization values of MMIPs also decreased compared to Fe_3_O_4_@SiO_2_, which were 25.58 and 79.65 emu/g, respectively. However, the decrease in the saturation magnetization of MMIPs did not adversely affect their magnetic separation ability. The separation of the sorbent from the solution using an external magnetic field could be completely and quickly accomplished in about 20 s.

## 4. Application of MMIPs to Natural Products

MMIPs have been widely used in the process of isolating active compounds contained in plants. They have advantages over other separation methods such as SPE and conventional MIPs in terms of the effectiveness of sample extraction from the magnetic particles and the selectivity of sample extraction produced by MIPs. Samples from plants usually contain a mixture of tens to hundreds of active compounds. MMIPs can be used to increase effectiveness in the separation of the target analyte compound from the matrix contained in the plant. In addition, the use of MMIPs in the process of isolating active compounds from plants has advantages in terms of the process because MMIPs can be applied in large-scale samples [60,99,100]. Table 4 describe some articles related to the application of MMIPs in the determination of active compounds from natural products.

### 4.1. Alkaloids

Alkaloids are secondary metabolites found in plants with properties that have been widely used in such areas as fever reduction and antimicrobial, analgesic, and insect attractant or repellent fields. One alkaloid that has many medicinal properties is harmaline, (1-methyl-7-methoxy-3,4-dihydro-β-carboline), an indole alkaloid found in various medicinal plants; one of which is *Peganum harmala*. Harmaline has properties as an anticoagulant, monoamine oxidase inhibitor, in boosting immunity, and as an antiviral and antiparasitic. Sadegh et al. [97] extracted harmaline from *P. harmala* by dispersive solid-phase micro extraction (DSPME) using MMIPs; this is an effective and selective method for extracting harmaline, synthesized using a sol–gel polymerization method with functionalized superparamagnetic core-shell nanoparticles. MMIP preparation begins with the synthesis of Fe_3_O_4_ MNPs. Fe_3_O_4_ MNPs were synthesized by means of FeCl_3_⋅6H_2_O (0.02 mol) and FeCl_2_⋅4H_2_O salt (0.01 mol) added to 150 mL of deionized water and then stirred thoroughly at 1000 rpm. Next, the solution was deoxygenated with nitrogen gas, and during this process, the temperature was raised to 60 °C. After 30 min, 20 mL of ammonia was added to the solution, then stirred for 150 min. At the end of the reaction, the MNP precipitate was removed from the reaction mixture with a magnet and washed with deionized water and ethanol. The resulting nanoparticles were dried in an oven at 60 °C. The next step is the synthesis of SiO2 coated Fe_3_O_4_ NPs. To a mixture of ethanol and deionized water (4:1), 200 mg Fe_3_O_4_ nanoparticles was added, and then sonicated in a water bath. Then, TEOS (2 mL) and ammonia (4 mL) were added to the solution. The reaction continued for 24 h at room temperature. The final product was separated by a magnetic field and washed several times by deionization water and ethanol and, finally, the sample was placed in an oven at 60 °C. The last step is the synthesis of the MMIP (Fe_3_O_4_@SiO_2_-MIP). Fe_3_O_4_@SiO_2_-NH_2_-MMIP nanoparticles were synthesized by dissolving 2 g of Fe_3_O_4_@SiO_2_ nanoparticles in 350 mL of double distilled water and ethanol (1:1). Then, 2 mL of APTMS was added to the mixture and refluxed overnight at 90 C. The obtained solid was then washed with ethanol and double distilled water to pH = 7. The Fe_3_O_4_@SiO_2_ nanoparticles modified with APTMS (Fe_3_O_4_@SiO_2_-APTMS) were separated and washed with double distilled water and ethanol to remove residual APTMS. After Fe_3_O_4_@SiO_2_-NH_2_ was synthesized, 0.073 g harmalin, 0.1 g Fe_3_O_4_@SiO_2_-NH_2_, and 0.114 g methacrylic acid were added to 75 mL ethanol and then sonicated in a water bath for 20 min. EGDMA was added to the mixture as a crosslinking agent (3.96 g) and AIBN as an initiator (0.06 g). Furthermore, nitrogen gas was used for the deoxygenation of the solution for 30 min, and the polymerization reaction was carried out overnight at 60 C. Finally, the obtained MMIP was then washed with methanol and acetic acid and dried in the oven. They evaluated the properties and morphology of the MMIPs obtained using FT-IR, TEM (transmission electron microscopy), SEM, and VSM, which showed that the obtained polymer had good selectivity, sensitivity, and recognition and suitable magnetic properties for harmaline extraction from plants. The extracted harmaline was then quantified and analyzed using HPLC-UV. The analytical method was also validated, and the limit of quantification (LOQ) and limit of detection (LOD) values were 0.526 and 0.158 ppb, respectively. The results of this study indicate that the DSPME technique using MMIPs as the sorbent is very effective and efficient and can even be used for preconcentration and extraction of harmaline from *P. harmala*.

Kang [68] published research on the selective extraction of quinolizidine alkaloids (oxymatrine and matrine) from *Sophora flavescens* using MMIPs. *Sophora flavescens* is a Chinese herbal plant, locally known as kushen, which has various pharmacological properties, with the main quinolizidine alkaloids contained being oxymatrine and matrine, which have anti-inflammatory, hepatoprotective, antinociceptive, and antitumor properties. The synthesis of MMIPs started with the synthesis of Fe_3_O_4_ and Fe_3_O_4_-SiO_2_, which were then dispersed in a mixture of 100 mL toluene and 3.35 mL MPS. This mixture was stirred for 12 h at 70 °C. The resulting Fe_3_O_4_-SiO_2_-MPS polymer was then washed with ultrapure water and dried under vacuum at 50 °C. The MMIP preparation was continued by reacting oxymatrine as a template (0.2 mmol) and acrylic acid as a functional monomer (1 mmol) in a flask filled with a mixture of toluene/acetonitrile (1:3). Then, the mixture was stirred for 5 h at room temperature (pre-polymerization step), and 100 mg of Fe_3_O_4_-SiO_2_-MPS previously prepared was dispersed into the solution for 10 min. EGDMA as the crosslinker (4 mmol) and AIBN as the initiator (0.3 mmol) were added to the mixture. The mixture was then heated at 60 °C for 24 h under a nitrogen atmosphere while stirring at 200 rpm. The polymer that was formed was collected using a magnet and the template eluted using a mixture of methanol/acetic acid (8:2). The polymer was then washed with methanol to remove acetic acid and dried under vacuum at 50 °C. The MMIP polymers were characterized using FT-IR, XRD, VSM, and TGA, indicating that the preparation was successful. The adsorption capacity of the MMIPs was analyzed using static and dynamic adsorption experiments and showed that the capacity factors for oxymatrine and matrine were 110.8 mg/g and 63.4 mg/g, respectively. This MMIP technique could be used as an effective method for the extraction and enrichment of oxymatrine and matrine in *S. flavescens* with oxymatrine and matrine extraction efficiencies of 80.21–89.15% and 85.33–95.28%, respectively.

### 4.2. Flavonoids

Flavonoids are secondary metabolites of polyphenols, found widely in plants as well as foods, and have various bioactive effects including anti-aging and antioxidant [108], anti-inflammatory and antiviral [109], and anticancer, cardioprotective, and anti-diabetic properties [110]. Flavonoids are polyphenol compounds with 15 carbon atoms arranged in the C6-C3-C6 configuration of a carbon skeleton containing two substituted benzene rings linked by aliphatic (three-carbon) chains [111]. They are present in all green plants, and therefore can be found in many plant extracts. They form a class of compounds that is widely expressed in nature. To date, more than 9000 flavonoids have been reported, and the daily requirements vary between 20 mg and 500 mg and are especially found in foods such as tea, red grapes, apples, onions, and tomatoes. The flavonoids found in plants contribute to the production of yellow, red, orange, blue, and purple colored pigments in fruit, flowers, and leaves [111].

Ma and Shi [93] determined quarcateganin in *Calendula officinalis* extract using MMIPs. Quarcateganin is a flavonol with the addition of six hydroxyl phenolic groups to the basic flavone structure and is the most common flavonoid in *C. officianalis*. Quarcateganin compounds have properties in medicine such as being anti-inflammatory and anticancer agents. Quarcateganin analysis is limited to the use of silica gel column chromatography or other traditional methods that have the disadvantages of long separation times, poor selectivity and efficiency, and being less environmentally friendly due to the use of many solvents. Therefore, Ma and Shi conducted a study using MMIPs with acrylamide as the functional monomer, EGDMA as the crosslinker, and acetonitrile as the porogen. In total, 250 mg Fe_3_O_4_@SiO_2_ nanoparticles was dispersed in 50 mL anhydrous toluene solution containing 5 mL MPS in a three-neck flask while stirring, followed by 15 min ultrasonic dispersion. The mixture was reacted for 24 h at 70 °C while refluxing in a nitrogen atmosphere. After that, the product (Fe_3_O_4_@SiO_2_-CH=CH_2_) was magnetically collected, and then freeze-dried for further use after washing with ethanol and deionized water. Next, 16 mg of quercetagetin was dissolved in 20 mL of acetonitrile in a three-neck flask, 21 mg of acrylamide was added to the solution and continuously stirred for 5 h to form a pre-polymer, and 100 mg of Fe_3_O_4_@SiO_2_-CH=CH_2_ was dried and then dispersed in the above solution. Then, 0.44 mL of EGDMA and 20 mg of AIBN were added for polymerization into solution and degassing for 15 min. Thereafter, the system was prepared at 65 °C for 24 h under mechanical stirring. In the above process, the nitrogen gas is purged continuously. After synthesis, MMIP was collected by external magnetic field and washed with methanol/acetic acid (8:2) mixture several times until no template was detected by HPLC-DAD. It was found that the sorbent showed a good adsorption capacity, and after being analyzed using the adsorption isotherm model, it was shown that the specific binding sites for quarcateganin were evenly formed on the surface of the MMIPs. The high selectivity and convenience shown by MMIPs in the determination of quarcateganin in *C. officinalis* extract confirms that this method is very easy and fast and can be used for the determination of quarcateganin in other herbal extracts. Therefore, the MMIP technique is very promising for use as an alternative method in determining active compounds in natural products.

### 4.3. Glycosides

Chen et al. [72] conducted a study on a selective and efficient method for the isolation of rhaponticin in Chinese patent medicine using the MMIPs technique. The Chinese patent medicine plant studied was rhubarb (*Rheum rhabarbarum*), which is believed to have properties for treating constipation, inflammation, and cancer [112] and in lowering blood sugar levels, obesity, and hyperlipidemia [113]. On the market, many Chinese patent medicines containing rhubarb are adulterated with unofficial rhubarb, which has different pharmacological activity from official rhubarb. Adulteration with unofficial rhubarb is usually used to save costs. Rhaponticin is one of the many ingredients found in unofficial rhubarb but not in official rhubarb. Therefore, in this study, MMIPs were used to analyze the content of rhaponticin in four complicated Chinese patent medicines. Synthesis of Fe_3_O_4_ magnetic particles was prepared by 15 mmol FeCl_3_·6H_2_O and 10 mmol FeCl_2_·4H_2_O dissolved in 80 mL deoxygenated water in a 250 mL three-neck flask. Then, a solution of ammonium hydroxide (28%) was added dropwise until a clear yellow color at 300 rpm under nitrogen gas was achieved. The solution was allowed to turn black. This solution was stirred for 30 min at 80 °C. After that, the black precipitate (Fe_3_O_4_ nanoparticles) was collected with a magnetic field, and then washed with deionized water until the pH was neutral. Then, 2 g Fe_3_O_4_ nanoparticles was added to 10 g polyethylene glycol (PEG) in 30 mL distilled water and stirred for 20 min, sonicate for 30 min to obtain a dispersed homogeneous solution. MMIPs were synthesized by suspension polymerization using PEG-Fe_3_O_4_ as a magnetic component, rhaponticin (1 mmol) as a template, acrylamide (6 mmol) as a functional monomer, styrene (79.6 mmol) as a monomer copolymer, EGDMA (30 mmol) as a crosslinker, AIBN (0.6 mmol) as an initiator, and DMSO (10 mL) as a porogen. The polymerization process took place under nitrogen protection at 70 °C for 24 h. The resulting MMIP polymer was physically characterized using FT-IR, XRD, SEM, and VSM. The selectivity of the MMIPs was investigated using resveratrol and kyrenol, which have chemical structures very similar to that of rhaponticin. The extraction process began with 200 mg of pulverized Chinese patent medicine mixed with 10 mL of methanol and sonicated for 20 min. The extract was then filtered and evaporated under nitrogen gas at room temperature. MMIPs (40 mg) were added to the residue and dissolved in 1 mL acetonitrile/methanol (9:1). The final residue was then analyzed using high performance liquid chromatography-ultraviolet (HPLC-UV). The results of the rhaponticin content of four Chinese patent medicinal samples were 11.84, 3.35, 4.47, and 7.57 ppm with percentage recoveries of 77.82–91.00%. The results of this study indicate that MMIPs can be applied to the determination and selective pre-concentration of rhaponticin in medicinal plants.

### 4.4. Polyphenols

Polyphenols are natural compounds in plants that have many health benefits. In the body, polyphenols act as antioxidants that can reduce the risk of various diseases. The benefits of these polyphenols can be obtained by eating healthy foods. Various studies state that polyphenols are useful in preventing damage to body cells due to free radicals and by boosting the immune system. These free radicals are formed naturally as a result of metabolic processes. Polyphenols also have other benefits, such as preventing cardiovascular disease, lowering blood sugar, lowering the risk of cancer, improving memory, and maintaining digestive health. Gu [60] conducted a study on the isolation of one polyphenol, chlorogenic acid, from the traditional Chinese medicinal plant honeysuckle (*Lonicera caprifolium*) using surface-imprinted magnetic nanoparticles using the water–oil–water multiple emulsion suspension polymerization method. Magnetic Fe_3_O_4_ nanoparticles were prepared by reacting 15 mmol FeCl_3_·6H_2_O in 80 mL deionized water. The solution was then deoxygenated with nitrogen gas for 20 min. FeCl_2_·4H_2_O (10 mmol) was added to the solution and mixed at 400 rpm at 35 °C. Ammonia (50 mL) was added dropwise and the solution turned black. This black solution was then heated in a water bath at 60 °C for 1 h. The Fe_3_O_4_ formed was collected using a magnet and washed with deionized water to neutral pH. The MIPs were prepared by reacting 0.15 mmol AIBN (initiator) with 5 mmol trimethylolpropane trimethacrylate (TRIM) (crosslinker), and then adding one drop of OA (emulsifier) in 1.5 mL toluene and mixing until homogeneous (oil phase). Fe_3_O_4_ nanoparticles (100 mg) were then slowly added to this oil phase. The mixture was then stirred for 10 min and ultrasonicated for 5 min to form a water–oil inverse emulsion. Then, 3 mmol of methacrylic acid (functional monomer) and 0.25 mmol of chlorogenic acid (template) were dissolved in 12.5 mL of 70% ethanol and hydroxyethyl cellulose (HEC) separately dissolved in 12.5 mL deionized water. These two solutions are mixed to form the aqueous phase, and the water–oil inverse emulsion containing Fe_3_O_4_ core was added dropwise. This mixture was then infused with nitrogen gas for 20 min to form a water–oil–water multiple emulsion. After that, polymerization was carried out at 70 °C for 12 h. The encapsulation efficiency of the nanoparticles produced in this study was 19.3 wt%. The magnetic MIPs that were produced showed good selectivity and specific binding sites for chlorogenic acid molecules; the capacity factor of the MIPs was three times greater than that of Non-Imprinted Polymers (NIPs). Magnetic MIPs also showed high selectivity compared to caffeic acid and this was six times higher than that of NIPs. The magnetic MIPs generated in this study were reused and regenerated four times; the fifth cycle still recovered up to 78.85% of that seen in the first binding. The surface-imprinted magnetic nanoparticles via water-in-oil-in-water multiple emulsion suspension polymerization showed great potential in the separation of chlorogenic acid from traditional Chinese medicines.

### 4.5. Terpenes

Liang [100] conducted a study on the synthesis of MMIPs to effectively extract sibiscoside from *Sibiraea angustata* using the surface imprinting polymerization technique. Magnetic core Fe_3_O_4_ was prepared using the solvothermal reaction method by dissolving FeCl_3_·6H_2_O (13.50 g, 0.05 mol) in ethylene glycol, to which sodium acetate was then added and autoclaved for 6 h at 200 °C. The magnetic core was then modified with SiO_2_ to form Fe_3_O_4_@SiO_2_. The MMIPs were prepared using an acrylamide monomer and a sibiscoside template dissolved in toluene. Then a magnetic core-shell was added, which had previously been modified using surface imprinting polymerization by adding 3-aminpropyltriethoxylsilane (APTES). Then, EGDMA and AIBN were added and sonicated for 15 min before being incubated on a shaker for 24 h at 60 °C. The resulting MMIP polymers were characterized by SEM, TEM, FT-IR, and TGA. In addition, an evaluation of the MMIP adsorption capacity forsibiscoside was also carried out, providing MIP and NIP values of 6.0 and 2.43 mg/g, respectively.

## 5. Conclusions

The isolation of compounds from natural plants using MMIPs can be a good choice as the technique provides good yields and the process of releasing the target compound from the sorbent can be carried out more quickly and efficiently with the help of an external magnet. However, several essential factors must be considered when obtaining MMIPs with high adsorption capacity and selectivity. The effectiveness of MMIPs depends on the magnetic core-shell particle size and distribution. Factors such as reaction time, molar ratio of FeCl_3_ and protective agents, initial concentration of FeCl_3_, pH, and reaction temperature should be optimized as they affect the particle size and shape of the magnetic core-shell. The particle size and distribution of the magnetic core-shell have important influences in determining the effectiveness of MMIPs. The co-precipitation method is the synthesis method that produces a high yield of magnetic core-shell and involves lower costs than the solvothermal method, even though it produces a more irregular particle size distribution. In addition, protective agents in the modification of the magnetic core-shell also affect the stability of the MMIPs bonds.

## 6. Further Perspectives

With some considerations regarding MMIP synthesis, much effort should be devoted to exploring future MMIP by following the directions below:

1. In the development of MMIP synthesis, the choice of shell material (protective) must consider the properties of other MMIP components, such as templates and functional monomers.
2. Further research is needed on the optimum conditions for coating the magnetite core with the shell so that it will be a breakthrough to obtaining a magnetic core-shell that has good resistance and stability.
3. There are many methods of synthesizing MNPs; this could be an opportunity for exploring further methods to try new techniques in MMIP synthesis.

## Figures and Tables

**Table 1:** Various polymerization techniques for molecularly imprinted polymer synthesis for natural products.

Type of Polymerization	Sample	Template Molecule	Functional Monomer	Crosslinker	Initiator	Porogen Solvent	Ref.
Bulk	*Rosmarinus officinalis* L.	Rosmarinic Acid	4-Vinyl pyridine	EGDMA	AIBN	DMSO	[31]
	*Plectranthus scutellarioides*	Quercetin	Acrylamide	EGDMA	AIBN	MeOH	[32]
	*Gracinia yunnanensis* Hu.	Oblongiofolin C	Acrylamide	EGDMA	AIBN	DMSO	[33]
	*Broccoli* (*Botrtis italica* L.)	Sinapic Acid	4-Vinyl pyridine	EGDMA	N/A	DMSO	[34]
	*Chicory herb* (*Chicorium intybus* L.)	Chicoric Acid	4-Vinyl pyridine	EGDMA	AIBN	DMSO	[35]
	*Alkana tinctoria* Roots	Shikonin	2-Diethylaminoethyl methacrylate	EGDMA	ABDV	CHCl_3_	[36]
	*Catharanthus roseus*	Catharanthine	Methacrylic Acid	EGDMA	AIBN	ACN	[37]
	*Liquorice roots* (*Glycirrhiza glabra*)	Glycyrrhizic Acid	2-Hydroxyethylmetacrylate	EGDMA	AIBN	DMF	[38]
In situ	*Radix Salviae Miltiorrhizae*	Propyl Gallate	4-Vinyl pyridine	EGDMA	AIBN	EtOAc	[39]
	*Sophorae flavescentis* Ait	Matrine	Methacrylic Acid	EGDMA	AIBN	Toluene	[40]
Precipitation	*Carthamus tinctorius* L.	Myricetin	4-Vinyl pyridine	EGDMA	AIBN	MeOH/ACN (1:2 *v*/*v*)	[41]
	*Sophora moorcroftiana*	Matrine and Oxymatrine	Methacrylic Acid	EGDMA	AIBN	ACN	[42]
	*Chrysanthemum morifolium* Ramat	Luteolin	Acrylamide	EGDMA	AIBN	ACN/DMSO (19:1 *v*/*v*)	[43]
	*Curcuma longa* L.	Curcumin	Methacrylic Acid	Divinylbenzene	AIBN	ACN/Toluene (3:1 *v*/*v*)	[44]
	*Sophora flavescens* Aiton	Matrine and Oxymatrine	Methacrylic Acid	Divinylbenzene	AIBN	ACN/Toluene (3:1 *v*/*v*)	[45]
	*Salicornia herbacea* L.	Phenolic Acid	Methacrylic Acid	EGDMA	AIBN	Alcohol/Water (9:1 *v*/*v*)	[46]
	*Rhizoma homalomenae*	Protocatechuic Acid	Acrylamide	EGDMA	AIBN	ACN	[47]
	Traditional Chinese Medicine	Podophylotoxin	Acrylamide	EGDMA	AIBN	ACN	[48]
	*Andrographis paniculata*	Andrographolide	Acrylamide	EGDMA	AIBN	ACN/Toluene (3:1 *v*/*v*)	[49]
Emulsion	*Spina gleditsiae*	Quercetin	4-Vinyl pyridine	Divinyl Benzene	AIBN	Water-THF	[50]
Suspension	Vegetable Extract	Polydatin	4-Vinyl pyridine	EGDMA	AIBN	Water/MeOH	[51]
	*Sophora flavescens* Ait	Matrine	Methacrylic Acid	EGDMA	AIBN	CHCl_3_	[52]

Abbreviations: ACN, acetonitrile; AIBN, 2,2′-azobisisobutyronitrile; ABDV, 2,2′-azobis(2,4-dimethylvaleronitrile); CHCl_3_, chloroform; DMF, dimethylformamide; DMSO, dimethyl sulfoxide; EGDMA, ethylene glycol dimethylacrylate; EtOAc, ethyl acetate; MeOH, methanol; THF, tetrahydrofuran.

**Table 2:** Factors affecting particle size and shape of magnetic core.

Factor	Effect [84]
Reaction time	Shorter reaction time, smaller size of nanoparticles [84]
Molar ratio of FeCl_3_ and protective agents	Higher molar ratio, smaller size of nanoparticles [84]
Initial concentration of FeCl_3_	Higher concentration, larger size of nanoparticles [84]
pH	Higher pH value, smaller size of nanoparticles [84]
Reaction temperature	Higher temperature, larger size of nanoparticles [85]

**Table 3:** Physical characterization of MMIPs.

Physical Characterization	Objective
Brunauer–Emmet–Teller (BET)	To analyze the porosity and specific surface area [91]
Fourier-transform infrared (FT-IR)	To determine the functional group of the molecular structure and the presence of Fe-O bond stretching vibration at 590 cm^−1^ [92]
Thermogravimetric analysis (TGA)	To analyze thermal stability and composition of a material [68]
Scanning electron microscopy (SEM)	To confirm molecule size distribution and morphology [93]
Vibration sample magnetometer (VSM)	To measure the magnetic separation ability of MMIPs, based on the relationship between magnetization and magnetic field strength [93]
X-ray diffraction (XRD)	To analyze the diffraction spectroscopy of materials [84]

**Table 4:** Application of MMIPs in natural plants.

Compound Group	Type of Polymerization	Sample	Analyte	Magnetic Materials	Monomer; Crosslinker; Initiator; Template	Yield/Purity (Y%/P%) and Adsorption Capacity (AC)	Ref.
Alkaloid	Precipitation Polymerization	*Sophora flavescens* Root	Quinolizidine Alkaloids	Fe_3_O_4_@SiO_2_@MPS	Acrylamide 1.0 mmol; EGDMA 4.0 mmol; AIBN 0.3 mmol; Oxymatrine 0.2 mmol	Y: 80.21–89.15% and 85.33–95.28% AC: 110.8 and 63.4 mg/g	[68]
	Sol–Gel Polymerization	*Peganum harmala extract*	Harmaline	Fe_3_O_4_@SiO_2_	Methacrylic Acid 1.3 mmol; EGDMA 18.6 mmol; AIBN 0.4 mmol; Harmaline 0.3 mmol	Y: >90% AC: 45.31 mg/g	[97]
	Surface Imprinting Polymerization	*Macleaya cordata*	Chelerythrine	Fe_3_O_4_@SiO_2_@MPS	Methacrylic Acid 0.04 mmol; EGDMA 2.0 mmol; AIBN 1.2 mmol; Chelerythrine 0.1 mmol	Y: not mentioned in the article AC: 7.96 mg/g	[65]
Flavonoid	Precipitation Polymerization	*Rhododendron species*	Farrerol, Taxifolin, Kaempferol, Hyperin	Fe_3_O_4_@SiO_2_-GO@MPS	4-Vinylpyridine 4.0 mmol; EGDMA 20 mmol; AIBN 0.1 mmol; Farrerol 0.1 mmol	Y: not mentioned in the article AC: 10.04–20.66 mg/g	[69]
	Surface Imprinting Polymerization	*Citrus reticulata Blanco*	Hesperitin	Fe_3_O_4_@SiO_2_@MPS	N-Isopropylacrylamide 0.078 mmol; EGDMA 0.78 mmol; AIBN 0.06 mmol; Hesperetin 0.026 mmol	Y: 90.5–96.9% AC: 16.648 mg/g	[101]
	Surface Imprinting Polymerization	Apple Samples	Kaempferol	Fe_3_O_4_@SiO_2_	Acrylamide 1.0 mmol; EGDMA 6.0 mmol; AIBN 0.2 mmol; Kaempferol 0.2 mmol	Y: not mentioned in the article AC: 3.84 mg/g	[98]
	Suspension Polymerization	Green Tea	Rutin	Fe_3_O_4_	Methacrylic Acid 4.0 mmol; EGDMA 25.0 mmol; AIBN 1.0 mmol; Rutin 0.5 mmol	Y: not mentioned in the article AC: 2.43 mg/g	[102]
	Precipitation Polymerization	*Larix griffithiana*	Dihydro-quercetin	Fe_3_O_4_@SiO_2_	4-Viniylpyridine 0.065 mmol; EGDMA 0.41 mmol; AIBN 0.15 mmol; Dihydroquercetin 0.016 mmol	Y: 76.64–101.8% AC: 7.56 mg/g	[103]
Glycoside	Suspension Polymerization	Chinese Patent Medicines	Rhaponticin	Fe_3_O_4_	Acrylamide 6.0 mmol; EGDMA 30.0 mmol; AIBN 0.6 mmol; Rhaponticin 1.0 mmol	Y: 77.82–91.00% AC: not mentioned in the article	[72]
Polyphenol	Precipitation Polymerization	*Homalomena occulta*, *Cynomorium songaricum*	Protocatechuic Acid (PA)	Fe_3_O_4_@SiO_2_-CH=CH2	Acrylamide 5.0 mmol; EGDMA 30.0 mmol; AIBN 0.6 mmol; PA 1.0 mmol	Y: 86.3–122% AC: not mentioned in the article	[63]
	Surface Imprinting Polymerization	Fruit Juice (apple, pineapple, orange, peach juice)	Protocatechuic Acid (PA)	Fe_3_O_4_@SiO_2_@MPS	4-Vinylpyridine 1.0 mmol; EGDMA 5.0 mmol; AIBN 0.1 mmol; PA 0.25 mmol	Y: 92–107% AC: 7.5 mg/g	[104]
	Surface Imprinting Polymerization	Wine	Rhapontigenin (resveratrol dummy analogues)	Fe_3_O_4_@SiO_2_@MPS	Acrylamide 0.5 mmol; EGDMA 3.0 mmol; AIBN 0.1 mmol; Rhapotingenin 0.1 mmol	Y: 79.3–90.6% AC: 5.33 mg/g	[105]
	Surface Imprinting Polymerization	*Syxigium aromaticum*	Protocatechuic Acid (PA)	Fe_3_O_4_@SiO_2_	4-Vinylpyridine 1.0 mmol; EGDMA 5.0 mmol; AIBN 0.1 mmol; PA 0.25 mmol	Y: 29.3 µg/g extract AC: 11.9 mg/g	[99]
	Suspension Polymerization	*Traditional Chinese Medicine (TCM)*	Chlorogenic Acid	Fe_3_O_4_@SiO_2_@MPS	Methacrylic Acid 3.0 mmol; TRIM 5.0 mmol; AIBN 0.15 mmol; Chlorogenic Acid 0.25 mmol	P: 80.58% AC: 5.07 mg/g	[60]
	Surface Imprinting Polymerization	*Taraxacum mon-golicum* Hand.-Mazz	Caffeic Acid	Fe_3_O_4_@SiO_2_@MPS	4-Vinylpyridine 0.2 mmol and 2-(Dimethylamino) Ethyl Methacrylate (DMA) 0.2 mmol; EGDMA 2.0 mmol; AIBN 0.1 mmol; Caffeic Acid 0.1 mmol	Y: 90.47–98.97% AC: 11.5 mg/g	[106]
	Surface Imprinting Polymerization	*Malus doumeri* (Bois) A.	Phloridzin	Fe_3_O_4_@SiO_2_@NH_2_	Fe_3_O_4_@SiO_2_@NH_2_ 0.13 mmol; EGDMA 0.85 mmol; AIBN 0.05 mmol; Phloridzin 0.08 mmol	Y: 81.45–90.27% AC:	[107]
Terpene	Surface Imprinting Polymerization	*Sibiraea angustata*	Sibiskoside	Fe_3_O_4_@SiO_2_	4-Vinyl Benzoic Acid 0.2 mmol; EGDMA 1.0 mmol; AIBN 0.12 mmol; Sibiskoside 0.05 mmol	Y: 6.0 mg/g extract AC: 13.75 mg/g	[100]

Abbreviation: AC, adsorption capacity; AIBN, 2,2′-azobisisobutyronitrile; EGDMA, ethylene glycol dimethylacrylate; P, purity; PA, protocatechuic acid; TRIM, trimethylolpropane trimethacrylate; Y, yield.

## Data Availability

Not applicable.

## Abbreviations

AC	Adsorption capacity
ACN	Acetonitrile
ABDV	2,2’-Azobis(2,4-dimethylvaleronitrile)
AIBN	2,2’-Azobisisobutironitrile
APTES	(3-Aminopropyl)triethoxysilane
BET	Brunauer-Emmet-Teller
CHCl3	Chloroform
DMA	2-(Dimethylamino) Ethyl Methacrylate
DMF	Dimethylformamide
DMSO	Dimethyl Sulfoxide
DSPME	Dispersive Solid-Phase Micro Extraction
EGDMA	Ethylene Glycol Dimethylacrylate
EtOAc	Ethyl Acetate
Fe_3_O_4_	Ferrosoferric Oxide
FT-IR	Fourier-Transform Infrared
HEC	Hydroxyethyl Cellulose
HPLC-UV	High Performance Liquid Chromatography–Ultraviolet
JCPDS	Joint Committee on Powder Diffraction Standards
LOD	Limit of Detection
LOQ	Limit of Quantification
MeOH	Methanol
MIPs	Molecularly Imprinted Polymers
MMA	Methyl Metacrylate
MMIP-SPE	Magnetic Molecularly Imprinted Polymer–Solid Phase Extraction
MMIPs	Magnetic Molecularly Imprinted Polymers
MNIPs	Magnetic Non-Imprinted Polymers
MNPs	Magnetic Nanoparticles
MPS	3-(Trimethoxysilyl) Propyl Methacrylate
NIPs	Non-Imprinted Polymers
OA	Oleic Acid
P	Purity
PEG	Polyethylene Glycol
PCMS	Polychloromethylstyrene
PA	Protocatechuic Acid
SEM	Scanning Electron Microscopy
SiO_2_	Silicon Oxide
SPE	Solid Phase Extraction
TEM	Transmission Electron Microscopy
TEOS	Tetraethyl Orthosilicate
TGA	Thermogravimetric Analysis
THF	Tetrahydrofuran
TRIM	Trimethylolpropane Trimethacrylate
VSM	Vibration Sample Magnetometer
XRD	X-Ray Diffraction
Y	Yield
